# Serial deep gray nuclear DTI changes in Parkinson’s disease over twelve years

**DOI:** 10.3389/fnagi.2023.1169254

**Published:** 2023-06-20

**Authors:** Yao-Chia Shih, Leon Qi Rong Ooi, Hui-Hua Li, John Carson Allen, Septian Hartono, Thomas Welton, Eng-King Tan, Ling Ling Chan

**Affiliations:** ^1^Department of Diagnostic Radiology, Singapore General Hospital, Singapore, Singapore; ^2^Duke-NUS Medical School, Singapore, Singapore; ^3^Graduate Institute of Medicine, Yuan Ze University, Taoyuan City, Taiwan; ^4^Department of Neurology, National Neuroscience Institute, Singapore, Singapore; ^5^Health Services Research Unit, Singapore General Hospital, Singapore, Singapore

**Keywords:** Parkinson’s disease, basal ganglia, diffusion tensor imaging (DTI), longitudinal, neurodegeneration

## Abstract

**Background:**

Deep gray nuclear pathology relates to motor deterioration in idiopathic Parkinson’s disease (PD). Inconsistent deep nuclear diffusion tensor imaging (DTI) findings in cross-sectional or short-term longitudinal studies have been reported. Long-term studies in PD are clinically challenging; decade-long deep nuclear DTI data are nonexistent. We investigated serial DTI changes and clinical utility in a case-control PD cohort of 149 subjects (72 patients/77 controls) over 12 years.

**Methods:**

Participating subjects underwent brain MRI at 1.5T; DTI metrics from segmented masks of caudate, putamen, globus pallidus and thalamus were extracted from three timepoints with 6-year gaps. Patients underwent clinical assessment, including Unified Parkinson Disease Rating Scale Part 3 (UPDRS-III) and Hoehn and Yahr (H&Y) staging. A multivariate linear mixed-effects regression model with adjustments for age and gender was used to assess between-group differences in DTI metrics at each timepoint. Partial Pearson correlation analysis was used to correlate clinical motor scores with DTI metrics over time.

**Results:**

MD progressively increased over time and was higher in the putamen (*p* < 0.001) and globus pallidus (*p* = 0.002). FA increased (*p* < 0.05) in the thalamus at year six, and decreased in the putamen and globus pallidus at year 12. Putaminal (*p* = 0.0210), pallidal (*p* = 0.0066) and caudate MD (*p* < 0.0001) correlated with disease duration. Caudate MD (*p* < 0.05) also correlated with UPDRS-III and H&Y scores.

**Conclusion:**

Pallido-putaminal MD showed differential neurodegeneration in PD over 12 years on longitudinal DTI; putaminal and thalamic FA changes were complex. Caudate MD could serve as a surrogate marker to track late PD progression.

## Introduction

Idiopathic Parkinson’s disease (PD) is a progressive neurodegenerative disease with resting tremor, rigidity, and hypokinesia attributed to dopaminergic depletion due to neuronal loss in the substantia nigra (Obeso et al., 2000; Chan et al., 2007; McGregor and Nelson, 2019; Tan et al., 2020). Braak histopathological staging (Braak et al., 2003) supports that Lewy pathology in the pars compacta of the substantia nigra leads to downstream disruption and complex compensative remodeling in the basal ganglia-thalamocortical circuitry (McGregor and Nelson, 2019), and spreads to multiple cortical regions in the later pathological stages throughout PD progression. The caudate, putamen, globus pallidus and thalamus are major components of the basal ganglia-thalamocortical circuitry that play important roles in the integration of motor functions (Lewis and Barker, 2009), and are amongst the main neuromodulatory therapeutic targets to alleviate PD-related motor symptoms (DeLong and Wichmann, 2015). However, *in vivo* neuroimaging evidence of the dynamics of microstructural changes in the deep gray nuclei throughout various stages of PD progression is still lacking.

Diffusion tensor imaging (DTI) allows noninvasive measurement of random motion of water molecules in tissues and provides quantitative indices to describe tissue microstructural characteristics (Basser and Pierpaoli, 1996). Axonal mean diffusivity (MD) is typically increased on brain DTI studies in PD (Kamagata et al., 2013), whereas fractional anisotropy (FA) has been reported to be sensitive, even to early cortical microstructural changes in PD in the absence of brain atrophy on anatomical T1-weighted imaging (Karagulle Kendi et al., 2008; Tessa et al., 2008). Cross-sectional DTI studies in PD have investigated microstructural changes in the subcortical nuclei, midbrain, and nigrostriatal tracts (Chan et al., 2007; Péran et al., 2010; Du et al., 2011; Tan et al., 2015; Atkinson-Clement et al., 2017). Overall, nigral alterations were consistently found in PD patients (Chan et al., 2007; Tan et al., 2015; Atkinson-Clement et al., 2017) in contrast to the heterogenous findings of basal ganglia-thalamo integrity reported in other studies (Gattellaro et al., 2009; Péran et al., 2010), suggesting diverse disease presentations at different stages of PD.

There are few prospective longitudinal DTI studies tracking brain microstructural changes in relation to PD progression (Sarasso et al., 2021). These short-term studies investigated temporal microstructural profiles of deep gray nuclei, and their findings were mixed (Chan et al., 2016; Loane et al., 2016). Loane et al. (2016) failed to detect diffusion changes in the striatum over 19 months, while increased putaminal FA and MD were reported in PD patients over a longer period (Chan et al., 2016). Long-term follow-up studies in PD using DTI are limited due to inherent clinical challenges with elderly patient recruitment, nature of this chronic debilitating disease in the later stages, and MR scanner upgrades over time (Sarasso et al., 2021). Serial, objective *in vivo* neuroimaging study of PD progression across a decade is non-existent. These could help us elucidate and understand the microstructural changes, especially in the deep gray nuclei, with potential to act as biomarkers in clinical trials, since treatment strategies are different between the early and late stages of PD (Cilia et al., 2020).

The present study investigated serial DTI changes in PD in the caudate, putamen, globus pallidus, and thalamus at three timepoints, 6 years apart. We also correlated deep gray nuclear DTI metrics with clinical variables of disease duration and severity to explore the potential of DTI metrics in tracking PD progression. We hypothesized that complex temporal and spatial DTI trajectories exist in the basal ganglia-thalamocortical circuitry, which begin in the striatum and extend to the thalamic nuclei due to secondary neurodegeneration, in line with Braak staging (Braak et al., 2003).

## Materials and methods

### Study participants

This study was approved by the institutional ethics review board, and all participants provided informed consent before entering the study at each timepoint. Clinical and imaging data (total *N* = 295 brain MRI) were available for this longitudinal MRI data analysis. The case-control cohort (N_total_ = 149) at timepoint 1 (Chan et al., 2007) comprised 72 consecutive patients clinically diagnosed with PD by an experienced movement disorder neurologist according to the United Kingdom PD Brain Bank criteria, and 77 age- and gender-matched healthy controls (HC) (excluding subjects with a history of brain injury, and/or any neurologic or psychiatric disease). 97 and 49 participants returned for brain MRI at timepoints 2 and 3, respectively (Table 1), on an average, six and 12 years, respectively after their first scan. The number of participants at each timepoint and those excluded are detailed in the flow diagram (Supplementary Figure 1) under Supplementary material. The clinico-demographic information with regard to age, gender, age of disease onset, Unified Parkinson Disease Rating Scale Part 3 (UPDRS-III), Hoehn and Yahr (H&Y) staging of PD severity, and levodopa equivalent doses over the 12-year study period were tabulated in Table 1. To verify if incongruent sample sizes with differential subject dropout between groups influenced the statistical power of the full cohort analysis, we repeated the same statistical analyses for the subset cohort comprising only subjects who returned for all three-timepoint brain MRIs. Clinico-demographic data of the subset cohort are listed in Table 2.

**TABLE 1:** Clinico-demographic data (full cohort).

Time point	Baseline (1st)				The 6th year (2nd)				The 12th year (3rd)			
**Group**	**HC** **(*n* = 77)** **mean (SD)**	**PD** **(*n* = 72)** **mean (SD)**	**Equality of means**	***p*-value**	**HC** **(*n* = 51)** **mean (SD)**	**PD** **(*n* = 46)** **mean (SD)**	**Equality of means**	***p*-value**	**HC** **(*n* = 31)** **mean (SD)**	**PD** **(*n* = 18)** **mean (SD)**	**Equality of means**	***p*-value**
Age in years	61.6 (9.4)	63.6 (10.1)	*t* = 1.23	0.2153	68.3 (9.0)	69.7 (9.2)	*t* = 0.73	0.4662	71.6 (9.1)	74.1 (11.4)	*t* = 0.86	0.3926
Gender (F/M)	37/40	35/37	χ^2^ = 0.01	0.9456	23/28	24/22	χ^2^ = 0.24	0.6221	16/15	11/7	χ^2^ = 0.12	0.7289
Age of disease onset in years	N.A.	58.3 (10.6)	N.A.	N.A.	N.A.	58.5 (9.8)	N.A.	N.A.	N.A.	57.8 (10.5)	N.A.	N.A.
Disease duration in years	N.A.	5.2 (3.9)	N.A.	N.A.	N.A.	11.2 (3.9)	N.A	N.A.	N.A.	16.3 (3.1)	N.A.	N.A.
H&Y	N.A.	2.4 (0.5)	N.A.	N.A.	N.A.	2.8 (0.7)	N.A.	N.A.	N.A.	3.5 (1.2)	N.A.	N.A.
UPDRS III	N.A.	28.9 (6.9)	N.A.	N.A.	N.A.	34.3 (7.6)	N.A.	N.A.	N.A.	45.1 (13.4)	N.A.	N.A.
Levodopa equivalent doses (mg/day)	N.A.	438.2 (363.0)	N.A.	N.A.	N.A.	421.4 (281.5)	N.A.	N.A.	N.A.	401.1 (257.7)	N.A.	N.A.

F, female; HC, healthy control; H&Y, Hoehn and Yahr staging; M, male; N.A., not applicable; PD, Parkinson’s disease; UPDRS, United Parkinson Disease Rating Scale.

**TABLE 2:** Clinico-demographic data (subset cohort).

Timepoint	Baseline (1st)				The 6th year (2nd)				The 12th year (3rd)			
**Group**	**HC** **(*n* = 29)** **mean (SD)**	**PD** **(*n* = 15)** **mean (SD)**	**Equality of means**	***p*-value**	**HC** **(*n* = 29)** **mean (SD)**	**PD** **(*n* = 15)** **mean (SD)**	**Equality of means**	***p*-value**	**HC** **(*n* = 29)** **mean (SD)**	**PD** **(*n* = 15)** **mean (SD)**	**Equality of means**	***p*-value**
Age in years	59.0 (9.0)	62.7 (11.1)	*t* = 1.18	0.2429	65.5 (8.7)	68.8 (11.1)	*t* = 1.09	0.2828	70.7 (8.6)	74.1 (11.3)	*t* = 1.12	0.2705
Gender (F/M)	14/15	9/6	χ^2^ = 0.54	0.4605	14/15	9/6	χ^2^ = 0.54	0.4605	14/15	9/6	χ^2^ = 0.54	0.4605
Age of disease onset in years	N.A.	57.9 (10.8)	N.A.	N.A.	N.A.	57.9 (10.8)	N.A.	N.A.	N.A.	57.9 (10.8)	N.A.	N.A.
Disease duration in years	N.A.	4.8 (3.1)	N.A.	N.A.	N.A.	10.9 (3.2)	N.A.	N.A.	N.A.	16.3 (3.1)	N.A.	N.A.
H&Y	N.A.	2.4 (0.3)	N.A.	N.A.	N.A.	2.7 (0.5)	N.A.	N.A.	N.A.	3.5 (1.1)	N.A.	N.A.
UPDRS III	N.A.	27.1 (4.7)	N.A.	N.A.	N.A.	31.5 (6.4)	N.A.	N.A.	N.A.	42.7 (17.9)	N.A.	N.A.
Levodopa equivalent doses (mg/day)	N.A.	224.2 (197.5)	N.A.	N.A.	N.A.	306.7 (206.9)	N.A.	N.A.	N.A.	393.3 (267.8)	N.A.	N.A.

F, female; HC, healthy control; H&Y, Hoehn and Yahr staging; M, male; N.A., not applicable; PD, Parkinson’s disease; UPDRS, United Parkinson Disease Rating Scale.

### Data acquisition

All subjects underwent brain MRI using a standardized protocol on a 1.5T Siemens Avanto scanner (MAGNETOM, Erlangen, Germany), taking care to scan the patients in their “on” stage with head positions stabilized by foam supports and velcro straps to reduce motion. The scans were acquired in the axial plane, parallel to the anterior-posterior commissure line. The DTI scan was performed using the following parameters: repetition time (TR)/echo time (TE), 4300/90 ms; *b*-values, 0 and 800 s/mm^2^; diffusion directions, 12; iPAT factor, 2; averages, 4; slice number, 27; slice thickness, 4 mm without gap; in-plane resolution, 1.2 × 1.2 mm^2^; matrix, 192 × 192; field of view, 23 × 23 cm^2^. Adjustments to minimize differences in image reconstruction using different coil combination modes due to MRI instrumental upgrades over time are provided in Supplementary material. Other MR sequences included (1) structural T1-weighted magnetization-prepared rapid gradient-echo (TR/TE/TI 1300/4.7/600 ms), (2) T2-weighted (TR/TE 5700/89 ms) and (3) fluid attenuated inversion recovery (TR/TE/TI 9000/97/2500 ms), which were reviewed by an experienced neuroradiologist at each timepoint to exclude neuropathology in the regions of interest (ROIs) (i.e., tumor and large-vessel infarctions).

### Data analysis

#### DTI preprocessing

Diffusion tensor imaging preprocessing was conducted using the standard FSL (FMRIB software library)^1^ pipeline, including skull stripping (*BET* tool) and corrections of eddy-current induced field inhomogeneities and head motions (*eddy* tool version 6.0.1) (Andersson and Sotiropoulos, 2016). The b-matrix was rotated to obtain the correct orientation information before starting the *eddy* procedure. The *eddy* tool then estimated and corrected the effect of both eddy currents and head motion on the raw DTI data. After preprocessing, all DTI datasets were used to fit a diffusion tensor model (DeLong and Wichmann, 2015) that produced the DTI metrics, FA and MD. Secondary DTI metrics (i.e., axial diffusivity [AD] and radial diffusivity [RD]) were also computed.

#### Segmentation of deep gray nuclei

The four deep gray nuclei (caudate, putamen, globus pallidus, and thalamus) were segmented in both hemispheres via a semi-automated pipeline (Supplementary Figure 2). First, for each subject, individual FA and MD maps were registered to the T1-weighted image via a transformation matrix, which was derived from the co-registration between the b0 and T1-weighted images using a rigid body transformation. Second, the individual T1-weighted images were normalized to the Montreal Neurological Institute (MNI) 152 T1-weighted template through the Symmetric image Normalization (SyN) algorithm^2^ (Avants et al., 2008). Third, eight subcortical ROIs obtained from the Harvard-Oxford subcortical structures atlas (Desikan et al., 2006) in the MNI space were automatically projected onto the individual diffusion maps using the reverse transformation in the SyN algorithm. All segmented nuclei were reviewed to ensure no voxel bleeding into adjacent white matter or cerebrospinal fluid before sampling of DTI metrics.

#### Statistical analysis

All statistical analyses were conducted using SAS version 9.4, R version 3.6.3, and SPSS version 20. χ^2^ and Student t tests were performed to assess the similarity of gender and age between PD and control groups. One-way Analysis of Variance with the Tukey Honestly Significant Difference *post-hoc* test was applied to compare each of the H&Y staging, UPDRS-III, and levodopa equivalent doses in patients across the three timepoints. Extracted DTI metrics of left and right ROIs were averaged. A multivariate linear mixed-effects regression model with adjustments for age and gender was used to assess between-group differences in DTI metrics at each timepoint by testing group-by-timepoint interaction in the following equation:


$$Y_{N\,u\,c\,l\,e\,u\,s}=A\,g\,e+G\,e\,n\,d\,e\,r+T\,i\,m\,e\,p\,o\,i\,n\,t+G\,r\,o\,u\,p+G\,e\,n\,d\,e\,r$$

×T⁢i⁢m⁢e⁢p⁢o⁢i⁢n⁢t+G⁢r⁢o⁢u⁢p×T⁢i⁢m⁢e⁢p⁢o⁢i⁢n⁢t+G⁢e⁢n⁢d⁢e⁢r

×G⁢r⁢o⁢u⁢p+G⁢e⁢n⁢d⁢e⁢r×T⁢i⁢m⁢e⁢p⁢o⁢i⁢n⁢t×G⁢r⁢o⁢u⁢p

where Y_Nucleus_ is a vector comprising the values of the given DTI metric in each nucleus. Partial Pearson correlation adjusting for potential confounding factors of age, gender and levodopa equivalent dose was used to separately correlate disease duration, H&Y staging, and UPDRS-III scores with deep gray nuclear FA and MD. The above correlation analyses were performed using the datapoints across three timepoints from all participants in both full and subset cohorts. The Benjamini–Hochberg method was used to correct *p* values for multiple tests. The Hedges’g effect size test was used to examine the between-timepoint differences in H&Y staging and UPDRS-III scores and between-group differences in DTI metrics at each timepoint.

## Results

### Demographics

There were no significant differences in either age or gender between the patients and HC at any timepoint (Tables 1, 2). Significant differences in the H&Y staging (*F*_(2,133)_ = 17.650, *p* < 0.001) and UPDRS-III (*F*_(2,133)_ = 28.760, *p* < 0.001) were found across all timepoints in patients, and results of *post-hoc* analyses are detailed in Supplementary Table 1.

### Microstructural changes of deep gray nuclei across 12 years

**FIGURE 1:**
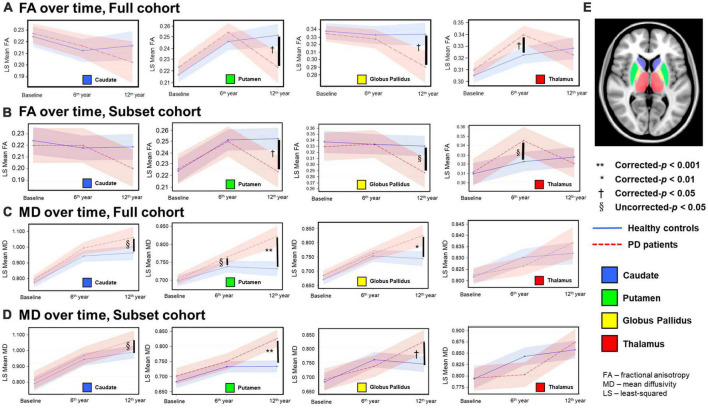
Temporal fractional anisotropy (FA) **(A,B)** and mean diffusivity (MD) **(C,D)** profiles in the four deep gray nuclei across 12 years. The profiles in rows **(A,C)** were obtained from all participants in the full cohort, whereas the profiles in the rows **(B,D)** were obtained from the subset cohort who completed all three-timepoint MRI examinations. The blue solid line indicates mean DTI metrics in healthy controls, whereas the red dotted line indicates mean DTI metrics in PD patients. Although between-group comparisons using the subset cohort dataset show marginal FA differences (uncorrected-*p* < 0.05) in the thalamus at the second timepoint and globus pallidus at the third timepoint **(B)**, the overall longitudinal DTI changes in all nuclei from both the full and subset cohorts are remarkably similar, indicating little censoring effect from subject dropout and/or incongruent sample sizes across 12 years in our results of the full cohort data. **(E)** Color coded labels of the four deep gray nuclear regions of interest (ROI) as defined in the Montreal Neurological Institute (MNI) 152 T1-weighted template.

For both full (Tables 3, 4) and subset (Tables 5, 6) cohorts at baseline, no significant differences in DTI metrics were found between patients and HC for any nucleus. Multivariate mixed-effects models revealed significant group-by-time interactions indicating temporal changes in the deep nuclear DTI metrics over time, even after correcting for multiple comparisons (Figure 1). In the full cohort at year six, thalamic FA was higher (*t* = −3.28, *p* = 0.0260) in patients than HC. Putaminal FA also rose in patients, but this was not different from HC. At year 12, putaminal and thalamic FA dropped, whereas pallido-putaminal MD continued to rise. Only putaminal (*t* = 3.38, *p* = 0.0189) and pallidal (*t* = 3.41, *p* = 0.0176) FA were lower in patients than HC; and MD was higher for both putamen (*t* = −5.19, *p* < 0.001) and globus pallidus (*t* = −4.00, *p* = 0.0023) as well in patients compared to HC. Both putaminal (*t* = −5.38, *p* < 0.001) and pallidal (*t* = −4.82, *p* < 0.001) RD, and putaminal AD (*t* = −4.36, *p* < 0.001) were higher in patients than HC at year 12. For the subset cohort, similar trends in DTI changes were found. At year 12, only putaminal FA decreased (*t* = 3.58, *p* = 0.0132), while MD increased in the putamen (*t* = −5.61, *p* < 0.001) and globus pallidus (*t* = −3.40, *p* = 0.0242) in patients compared to HC. Putaminal (*t* = −5.70, *p* < 0.001) and pallidal (*t* = −3.60, *p* = 0.132) RD, and putaminal AD (*t* = −4.76, *p* < 0.001) were also higher in patients than HC at year 12.

**TABLE 3:** Group comparisons of deep gray nuclear FA and MD at three time points 6 years apart (full cohort).

Deep gray nuclei	FA (ratio)						MD (10^–3^ mm^2^/s)					
	**HC** **mean (SD)**	**PD** **mean (SD)**	***t*-value**	***p*-value**	**Corrected *p*-value**	**Hedges’ g**	**HC** **mean (SD)**	**PD** **mean (SD)**	***t*-value**	***p*-value**	**Corrected *p*-value**	**Hedges’ g**
**1st**												
Caudate	0.223 (0.040)	0.226 (0.043)	**−**0.46	0.6472	N.S.	**−**0.0723	0.771 (0.082)	0.783 (0.091)	**−**0.73	0.4650	N.S.	**−**0.1388
Putamen	0.214 (0.024)	0.222 (0.025)	**−**1.45	0.1489	N.S.	**−**0.3267	0.687 (0.055)	0.697 (0.057)	**−**0.41	0.6840	N.S.	**−**0.1787
Globus pallidus	0.341 (0.040)	0.336 (0.044)	0.46	0.6444	N.S.	0.1191	0.655 (0.067)	0.677 (0.075)	**−**1.12	0.2637	N.S.	**−**0.3100
Thalamus	0.307 (0.021)	0.310 (0.021)	**−**1.05	0.2957	N.S.	**−**0.1429	0.795 (0.074)	0.805 (0.064)	**−**0.36	0.7169	N.S.	**−**0.1442
**2nd**												
Caudate	0.213 (0.031)	0.216 (0.039)	**−**0.55	0.5844	N.S.	**−**0.0857	0.948 (0.144)	1.005 (0.196)	**−**1.94	0.0545	N.S.	**−**0.3341
Putamen	0.247 (0.027)	0.255 (0.042)	**−**1.47	0.1442	N.S.	**−**0.2291	0.740 (0.046)	0.772 (0.099)	**−**2.23	0.0277	N.S.	**−**0.4218
Globus pallidus	0.329 (0.037)	0.325 (0.065)	0.63	0.5306	N.S.	0.0767	0.760 (0.066)	0.768 (0.112)	**−**0.60	0.5511	N.S.	**−**0.0882
Thalamus	0.322 (0.022)	0.338 (0.045)	**−**3.28	**0.0013**	**0.0260**	**−0.4592**	0.853 (0.064)	0.840 (0.100)	1.40	0.1630	N.S.	0.1566
**3rd**												
Caudate	0.220 (0.037)	0.193 (0.042)	1.67	0.0966	N.S.	0.6944	0.979 (0.175)	1.096 (0.159)	**−**2.43	0.0164	N.S.	**−**0.6907
Putamen	0.256 (0.041)	0.223 (0.026)	3.38	**0.0009**	**0.0189**	**0.9092**	0.739 (0.064)	0.840 (0.067)	**−**5.19	**<0.0001**	**<0.001**	**−1.5514**
Globus pallidus	0.329 (0.044)	0.282 (0.026)	3.41	**0.0008**	**0.0176**	**1.2216**	0.759 (0.100)	0.852 (0.078)	**−**4.00	**0.0001**	**0.0023**	**−1.0038**
Thalamus	0.327 (0.025)	0.316 (0.035)	0.77	0.4448	N.S.	0.3791	0.871 (0.060)	0.908 (0.087)	**−**1.10	0.2742	N.S.	**−**0.5214

Statistical significance is defined at corrected-*p* < 0.05 (marked in bold).

FA, fractional anisotropy; HC, healthy control; MD, mean diffusivity; N.S., not significant; PD, Parkinson’s disease.

**TABLE 4:** Group comparisons of deep gray nuclear radial diffusivity (RD) and axial diffusivity (AD) at three timepoints 6 years apart (full cohort).

DTI metric	RD (10^–3^ mm^2^/s)						AD (10^–3^ mm^2^/s)					
**Timepoint / Deep gray nuclei**	**HC** **mean (SD)**	**PD** **mean (SD)**	***t*-value**	***p*-value**	**Corrected *p*-value**	**Hedges’ g**	**HC** **mean (SD)**	**PD** **mean (SD)**	***t*-value**	***p*-value**	**Corrected *p*-value**	**Hedges’ g**
1st	***N* = 77**	***N* = 72**					***N* = 77**	***N* = 72**				
Caudate	0.685 (0.084)	0.695 (0.094)	**−**0.68	0.5007	N.S.	**−**0.1124	0.943 (0.089)	0.958 (0.094)	**−**0.79	0.4298	N.S.	**−**0.1640
Putamen	0.615 (0.052)	0.620 (0.057)	**−**0.11	0.9155	N.S.	**−**0.0918	0.833 (0.066)	0.849 (0.061)	**−**0.85	0.3988	N.S.	**−**0.2514
Globus pallidus	0.537 (0.067)	0.556 (0.078)	**−**0.74	0.4631	N.S.	**−**0.2620	0.892 (0.079)	0.920 (0.084)	**−**1.40	0.1647	N.S.	**−**0.3438
Thalamus	0.672 (0.070)	0.680 (0.063)	**−**0.24	0.8095	N.S.	**−**0.1199	1.040 (0.084)	1.054 (0.069)	**−**0.72	0.4729	N.S.	**−**0.1815
2nd	*N* = 51	*N* = 46					*N* = 51	*N* = 46				
Caudate	0.853 (0.140)	0.904 (0.192)	**−**1.77	0.0793	N.S.	**−**0.3060	1.139 (0.160)	1.207 (0.208)	**−**2.16	0.0325	N.S.	**−**0.3690
Putamen	0.648 (0.045)	0.674 (0.094)	**−**1.79	0.0763	N.S.	**−**0.3588	0.925 (0.055)	0.970 (0.115)	**−**2.85	0.0051	N.S.	**−**0.5077
Globus pallidus	0.627 (0.068)	0.641 (0.111)	**−**0.67	0.5071	N.S.	**−**0.1539	1.025 (0.073)	1.025 (0.114)	0.16	0.8748	N.S.	0.0000
Thalamus	0.712 (0.062)	0.695 (0.098)	1.68	0.0948	N.S.	0.2097	1.136 (0.072)	1.130 (0.107)	3.00	0.0033	N.S.	0.0665
3rd	*N* = 31	*N* = 18					*N* = 31	*N* = 18				
Caudate	0.888 (0.165)	0.998 (0.164)	**−**2.47	0.0147	N.S.	**−**0.6681	1.184 (0.192)	1.242 (0.245)	**−**2.26	0.0253	N.S.	**−**0.2727
Putamen	0.644 (0.066)	0.747 (0.070)	**−**5.38	**<0.0001**	**<0.001**	**−1.5265**	0.932 (0.071)	1.026 (0.070)	**−**4.36	**<0.0001**	**<0.001**	**−1.3307**
Globus pallidus	0.627 (0.098)	0.789 (0.276)	**−**4.82	**<0.0001**	**<0.001**	**−0.8827**	1.019 (0.102)	1.100 (0.101)	**−**2.98	0.0034	N.S.	**−**0.7969
Thalamus	0.724 (0.058)	0.762 (0.091)	**−**1.14	0.2581	N.S.	**−**0.5299	1.166 (0.072)	1.199 (0.084)	**−**1.10	0.2742	N.S.	**−**0.4310

Statistical significance is defined at corrected-*p* < 0.05 (marked in bold).

AD, axial diffusivity; N.S., not significant; HC, healthy control; PD, Parkinson’s disease; RD, radial diffusivity.

**TABLE 5:** Group comparisons of deep gray nuclear FA and MD at three time points 6 years apart (subset cohort).

Time Point / Deep gray nuclei	FA (ratio)						MD (10^–3^ mm^2^/s)					
	**HC (*N* = 29)** **mean (SD)**	**PD (*N* = 15)** **mean (SD)**	***t*-value**	***p*-value**	**Corrected *p*-value**	**Hedges’ g**	**HC (*N* = 29)** **mean (SD)**	**PD (*N* = 15)** **mean (SD)**	***t*-value**	***p*-value**	**Corrected *p*-value**	**Hedges’ g**
**1st**												
Caudate	0.214 (0.031)	0.208 (0.027)	0.45	0.6552	N.S.	0.2018	0.743 (0.043)	0.784 (0.071)	**−**0.69	0.4909	N.S.	**−**0.7597
Putamen	0.210 (0.023)	0.212 (0.022)	**−**0.21	0.8353	N.S.	**−**0.0882	0.676 (0.034)	0.706 (0.036)	**−**1.12	0.2681	N.S.	**−**0.8651
Globus pallidus	0.339 (0.038)	0.330 (0.044)	0.56	0.5753	N.S.	0.2244	0.656 (0.062)	0.675 (0.049)	**−**0.36	0.7220	N.S.	**−**0.3276
Thalamus	0.309 (0.021)	0.305 (0.023)	**−**0.17	0.8628	N.S.	**−**0.1844	0.776 (0.039)	0.796 (0.049)	**−**0.09	0.9276	N.S.	**−**0.4695
**2nd**												
Caudate	0.213 (0.033)	0.210 (0.044)	**−**0.24	0.8132	N.S.	**−**0.0810	0.929 (0.120)	0.972 (0.172)	**−**0.69	0.4944	N.S.	**−**0.3082
Putamen	0.249 (0.030)	0.244 (0.043)	0.17	0.8646	N.S.	0.1434	0.731 (0.051)	0.766 (0.084)	**−**1.06	0.2933	N.S.	**−**0.5475
Globus pallidus	0.333 (0.039)	0.332 (0.073)	**−**0.03	0.9740	N.S.	**−**0.0189	0.748 (0.115)	0.754 (0.074)	0.99	0.3276	N.S.	0.0582
Thalamus	0.322 (0.021)	0.337 (0.061)	**−**2.51	0.0142	N.S.	**−**0.3829	0.842 (0.045)	0.823 (0.110)	2.39	0.0192	N.S.	0.2590
**3rd**												
Caudate	0.219 (0.036)	0.193 (0.042)	1.99	0.0506	N.S.	0.6823	0.984 (0.175)	1.096 (0.159)	**−**2.16	0.0340	N.S.	**−**0.6595
Putamen	0.255 (0.040)	0.222 (0.026)	3.58	**0.0006**	**0.0132**	**0.9181**	0.737 (0.060)	0.842 (0.069)	**−**5.61	**<0.0001**	**<0.001**	**−1.6630**
Globus pallidus	0.329 (0.043)	0.283 (0.026)	3.08	0.0028	N.S.	1.2047	0.753 (0.090)	0.846 (0.076)	**−**3.40	**0.0011**	**0.0242**	**−1.0866**
Thalamus	0.328 (0.025)	0.313 (0.035)	0.78	0.4376	N.S.	0.5222	0.869 (0.060)	0.914 (0.087)	**−**1.00	0.3210	N.S.	**−**0.6414

Statistical significance is defined at corrected-*p* < 0.05 (marked in bold).

FA, fractional anisotropy; HC, healthy control; MD, mean diffusivity; N.S., not significant; PD, Parkinson’s disease.

**TABLE 6:** Group comparisons of deep gray nuclear radial diffusivity (RD) and axial diffusivity (AD) at three timepoints 6 years apart (subset cohort).

Timepoint / Deep gray nuclei	RD (ratio)						AD (10^–3^ mm^2^/s)					
	**HC (*N* = 29)** **mean (SD)**	**PD (*N* = 15)** **mean (SD)**	***t*-value**	***p*-value**	**Corrected *p*-value**	**Hedges’ g**	**HC (*N* = 29)** **mean (SD)**	**PD (*N* = 15)** **mean (SD)**	***t*-value**	***p*-value**	**Corrected *p*-value**	**Hedges’ g**
**1st**												
Caudate	0.661 (0.050)	0.707 (0.070)	**−**0.73	0.4652	N.S.	**−**0.8008	0.905 (0.039)	0.949 (0.071)	**−**0.59	0.5580	N.S.	**−**0.8477
Putamen	0.604 (0.036)	0.633 (0.032)	**−**0.88	0.3811	N.S.	**−**0.8353	0.815 (0.038)	0.856 (0.036)	**−**1.34	0.1850	N.S.	**−**1.0979
Globus Pallidus	0.541 (0.061)	0.567 (0.065)	**−**0.18	0.8587	N.S.	**−**0.4169	0.889 (0.071)	0.914 (0.052)	**−**0.17	0.8672	N.S.	**−**0.3829
Thalamus	0.654 (0.038)	0.673 (0.052)	**−**0.04	0.9700	N.S.	**−**0.4401	1.019 (0.044)	1.037 (0.047)	**−**0.12	0.9042	N.S.	**−**0.3998
**2nd**												
Caudate	0.836 (0.117)	0.869 (0.172)	**−**0.55	0.5674	N.S.	**−**0.2395	1.115 (0.125)	1.160 (0.188)	**−**0.84	0.4060	N.S.	**−**0.3020
Putamen	0.639 (0.051)	0.677 (0.083)	**−**1.03	0.3059	N.S.	**−**0.5986	0.914 (0.056)	0.953 (0.088)	**−**0.99	0.3251	N.S.	**−**0.5706
Globus pallidus	0.623 (0.075)	0.633 (0.128)	0.47	0.6396	N.S.	0.1042	1.016 (0.080)	1.013 (0.123)	1.65	0.1028	N.S.	0.0311
Thalamus	0.702 (0.044)	0.681 (0.114)	2.38	0.0197	N.S.	0.2801	1.123 (0.052)	1.103 (0.102)	2.22	0.0295	N.S.	0.2755
**3rd**												
Caudate	0.888 (0.165)	0.959 (0.226)	1.67	0.0293	N.S.	0.3786	1.185 (0.192)	1.242 (0.245)	**−**1.98	0.0517	N.S.	**−**0.2670
Putamen	0.931 (0.071)	1.026 (0.070)	**−**5.70	**<0.0001**	**<0.001**	**−1.3443**	0.931 (0.071)	1.026 (0.070)	**−**4.76	**<0.0001**	**<0.001**	**−1.3443**
Globus Pallidus	0.626 (0.088)	0.794 (0.284)	**−**3.60	**0.0006**	**0.0132**	**−0.9384**	1.019 (0.102)	1.100 (0.101)	**−**2.51	0.0142	N.S.	**−**1.0866
Thalamus	0.723 (0.057)	0.763 (0.095)	**−**1.06	0.2913	N.S.	**−**0.5561	1.166 (0.072)	1.199 (0.084)	**−**0.87	0.3857	N.S.	**−**0.4330

Statistical significance is defined at *p* < 0.05 (marked in bold).

AD, axial diffusivity; HC, healthy control; MD, mean diffusivity; N.S., not significant; PD, Parkinson’s disease; RD, radial diffusivity.

### Relationships with clinical variables

**FIGURE 2:**
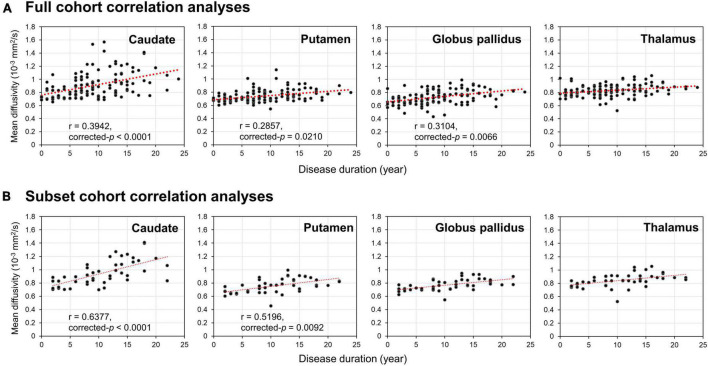
Results of Partial Pearson correlation analysis with adjustments for age, gender and levodopa equivalent dose. Correlations of disease duration with mean diffusivity of the four deep gray nuclei (caudate, putamen, globus pallidus, and thalamus) provided by the panel **(A)** full and **(B)** subset cohort data across 12 years.

Partial Pearson correlation analyses identified several modest but significant relationships between deep gray nuclear DTI metrics and clinical metrics after multiple testing corrections (Table 7). For the full cohort, MD in the caudate (*r* = 0.394, *p* < 0.0001), putamen (*r* = 0.286, *p* = 0.0210) and globus pallidus (*r* = 0.310, *p* = 0.0066) showed positive correlations with disease duration (Figure 2A). MD in the caudate additionally correlated with both H&Y staging (*r* = 0.276, *p* = 0.0300) and UPDRS-III scores (*r* = 0.305, *p* = 0.0108). For the subset cohort, MD in the caudate (*r* = 0.638, *p* < 0.0001) and putamen (*r* = 0.520, *p* = 0.0092) also showed positive correlations with disease duration (Figure 2B). In contrast, none of the nuclear FA measurements were associated with any clinical variable, for either cohort.

**TABLE 7:** The relationships between the deep gray nuclear DTI metrics and clinical measures described by Partial Pearson correlation analysis, controlling for age, gender, and levodopa equivalent dose (full and subset cohorts).

Clinical score	Disease duration			H&Y			UPDRS III		
**Deep gray nuclei**	**Coefficient r**	***p*-value**	**Corrected *p*-value**	**Coefficient r**	***p*-value**	**Corrected *p*-value**	**Coefficient r**	***p*-value**	**Corrected *p*-value**
**Full cohort**									
**FA (ratio)**									
Caudate	**−**0.1256	0.1559	N.S.	**−**0.1085	0.2209	N.S.	**−**0.1866	0.0342	N.S.
Putamen	0.1528	0.0838	N.S.	0.0875	0.3241	N.S.	0.0619	0.486	N.S.
Globus Pallidus	**−**0.2579	0.0032	N.S.	**−**0.0325	0.7143	N.S.	**−**0.0505	0.5696	N.S.
Thalamus	0.2223	0.0113	N.S.	0.0662	0.4555	N.S.	0.0301	0.7343	N.S.
**MD (10^– 3^ mm^2^/s)**									
Caudate	0.3942	**4.0 × 10^–^** ^6^	**9.6 × 10^–^** ^5^	0.2764	**0.0015**	**0.03**	0.3053	**4.7 × 10^–^** ^4^	**0.0108**
Putamen	0.2857	**0.001**	**0.021**	0.2111	0.0163	N.S.	0.1422	0.1078	N.S.
Globus pallidus	0.3104	**0.0003**	**0.0066**	0.0617	0.4871	N.S.	0.07	0.4308	N.S.
Thalamus	0.1801	0.0412	N.S.	0.0914	0.3031	N.S.	0.1454	0.1002	N.S.
**Subset cohort**									
**FA (ratio)**									
Caudate	**−**0.1616	0.3065	N.S.	**−**0.1807	0.252	N.S.	**−**0.3642	0.0177	N.S.
Putamen	0.1109	0.4845	N.S.	**−**0.042	0.7918	N.S.	0.0197	0.9016	N.S.
Globus pallidus	**−**0.2524	0.1067	N.S.	**−**0.0795	0.6166	N.S.	**−**0.0387	0.808	N.S.
Thalamus	0.0814	0.6083	N.S.	0.012	0.9399	N.S.	**−**0.0168	0.9161	N.S.
**MD (10^– 3^ mm^2^/s)**									
Caudate	0.6377	**6 × 10^–^** ^6^	**1.4 × 10^–^** ^4^	0.3479	0.024	N.S.	0.4166	0.0061	N.S.
Putamen	0.5196	**0.0004**	**0.0092**	0.357	0.0203	N.S.	0.1504	0.3417	N.S.
Globus pallidus	0.425	0.005	N.S.	0.164	0.2992	N.S.	0.0106	0.9471	N.S.
Thalamus	0.3967	0.0092	N.S.	0.1956	0.2146	N.S.	0.3077	0.4746	N.S.

Statistical significance is defined at corrected-*p* < 0.05 (marked in bold).

FA, fractional anisotropy; H&Y, Hoehn and Yahr staging; MD, mean diffusivity; N.S., not significant; PD, Parkinson’s disease; UPDRS, Unified Parkinson Disease Rating Scale.

## Discussion

The major strength of our study lies in this being a three-timepoint longitudinal imaging study spanning 12 years in an ageing case-control PD cohort. To our best knowledge, the present study is the longest follow-up DTI study on PD patients and age-matched HC (Yang et al., 2018; Sarasso et al., 2021), using a total of 295 brain MRI datasets acquired by a single DTI protocol on a 1.5T MRI scanner. Despite inherent limitations with elderly study participants, including debilitating co-morbidities resulting in subject dropouts over time, the temporal and spatial trajectories of nuclear DTI changes were remarkably similar for both the full and subset cohorts. Pallido-putaminal MD linearly increased as the disease evolved. FA changes were more complex. Only the caudate MD profile tracked the severity of motor disabilities in PD patients across the 12 years.

As the disease progressed into the late stage at year 12, both nuclear MD increases and FA decreases in patients suggest a loss of microstructural integrity and neuronal degeneration. These changes dominated in the putamen and globus pallidus, while relatively sparing the caudate and thalamus. The striatum and globus pallidus are spatially and functionally segregated as parallel units in the cortex-basal ganglia-thalamocortical circuitry (DeLong and Wichmann, 2015; McGregor and Nelson, 2019). The post-commissural putamen receives the bulk of the sensorimotor cortical glutamatergic afferents, while the globus pallidus serves as the main motor output nucleus to the thalamus. Molecular imaging studies in PD also showed that the progression rate of neuronal degeneration in the putamen is fastest in the posterior putamen, followed by the anterior putamen and caudate (Morrish et al., 1998; Nurmi et al., 2001). In addition, there is good corroborative *in vivo* 18F-DOPA PET and neuropathological immunohistochemical data of significant dopaminergic terminal depletion in the posterior/dorsal putamen by 5 years into clinical diagnosis (Nurmi et al., 2001; Kordower et al., 2013). Yet, abnormal pallido-putaminal DTI metrics were only found in our patients late into year 12. The above evidence suggests that, rather than nigral dopaminergic axonal degeneration, our DTI changes at year 12 may reflect cell loss within the sensorimotor units of the basal ganglia. Overall, our DTI findings appear to delineate a long-term microstructural striatopallidal neuronal degeneration secondary to chronic nigrostriatal denervation, and in line with Braak pathological staging (Braak et al., 2003). These findings also reveal somatotopic concepts of basal ganglia motor pathways and circuit dysfunction in PD (DeLong and Wichmann, 2015; McGregor and Nelson, 2019).

Our aging HC showed a modest rise in putaminal and thalamic FA across 12 years, which was not evident in the caudate or globus pallidus. In the first 6 years, while FA was not different between HC and patients in the putamen, it was higher in the thalamus in patients. A complex interplay of iron and levodopa effects (Rulseh et al., 2013; Xu et al., 2015; Du et al., 2018, 2022), and neuroadaptative responses in the basal ganglia-thalamocortical circuitry (Mole et al., 2016; Atkinson-Clement et al., 2017) may account for these complicated FA findings. *In vivo* MRI and post mortem studies have demonstrated higher iron content in the putamen and globus pallidus than the caudate in the normal adult brain, with higher aging-related accumulation consistently found in the putamen (Hallgren and Sourander, 1958; Bartzokis et al., 2007; Ramos et al., 2014; Xu et al., 2015). In contrast, iron levels in the thalamus are low, and remain low in spite of aging (Hallgren and Sourander, 1958; Bartzokis et al., 2007). Notably, high iron estimates have been previously shown to be positively correlated with high FA in the putamen in the healthy elderly (Pfefferbaum et al., 2010; Xu et al., 2015). Ferritin-bound iron is known to cause an artifactual rise in FA,(Rulseh et al., 2013) and the iron effect affects FA more than MD (Pfefferbaum et al., 2010; Rulseh et al., 2013; Xu et al., 2015). These findings from the literature support the argument that physiological iron accumulation in the putamen contributed to the FA rise in our elderly controls across 12 years.

A meta-analysis of cadaveric histochemical and MRI brain iron publications also showed pathological iron deposition in the putamen in PD, with similar evidence for globus pallidus and caudate only from MRI studies (Wang et al., 2016). The most recent literature evaluating nigral iron suggests that iron accumulation in PD is additionally affected by the type of antiparkinson medication (Du et al., 2018, 2022). Du et al. (2018, 2022) found that nigral iron accumulation plateaue by the middle stage of the disease, which they observed to also correspond to the end of the “honeymoon” phase of levodopa therapy (Cilia et al., 2020). These emerging data could explain the drop in putaminal FA beyond year six in patients, as they entered the later Braak stages. There is also molecular and neuropathological evidence of more rapid neurodegeneration in the dorsal than ventral putamen in PD (Morrish et al., 1998; Nurmi et al., 2001; Kordower et al., 2013). Manual segmentation of the anterior and posterior putamen have revealed spatially distinct putaminal DTI changes over 6 years (Chan et al., 2016). However, our automated masks with larger coverage would homogenize DTI metrics in distinct nuclear subregions, and such an approach could also influence statistical power of all our nuclear DTI measurements to differentiate patients and HC at the first two timepoints. Taken together, the combination of co-dependent biological processes and partial volume averaging by automated masks could explain, at least in part, the apparent lack of differences in regional FA and diffusivity metrics between patients and HC by year six.

Elevated thalamic FA at year six could be attributed to interim adaptative reorganization or selective neurodegeneration in the basal ganglia-thalamocortical circuitry before transition into the later Braak stages (Mole et al., 2016; Atkinson-Clement et al., 2017). The thalamus is a relay station and contains an abundance of myelinated crossing fibers between and within compartmentalized subnuclei (Battistella et al., 2017). In the “axonal dying back” model, the aggregation of α-synuclein proteins toxically causes selective axonal degeneration prior to neuronal death (Burke and O’Malley, 2013). Compensatory axonal sprouting in an aberrant neural network is believed to be contributed by mixed effects of intrinsic neuroplasticity and long-term therapeutic levodopa neuromodulation, with enhanced axonal packing (Arkadir et al., 2014; Herz et al., 2015; Mole et al., 2016; Atkinson-Clement et al., 2017). Increased FA has been reported in the thalamus-motor cortex tract and corticospinal tract in other DTI studies in PD (Mole et al., 2016; Atkinson-Clement et al., 2017). The FA rise in the thalamus in our patients at year six also supports the case for extranigral neuroplastic responses to altered pallidothalamic activity in PD (Mole et al., 2016).

Our temporal striatopallidal MD changes significantly correlated with disease duration, while FA changes failed to correlate with any clinical variable. This is congruent with the notion that MD estimates neuronal density in gray matter (Zhang and Burock, 2020), while FA is better suited to measure microstructural integrity of highly orientated tissues. Complicated motor comorbidities such as postural instability, freezing of gait, and falls are found in advanced stages of PD (Kalia and Lang, 2015), and molecular imaging studies suggest that abnormal caudate function relates to these symptomatologies (Zhang L. et al., 2016; Kim et al., 2018; Bohnen et al., 2019). These studies lend support to caudate MD sensitivity to heterogeneous comorbidities contributing to clinical motor dysfunctions in PD, and its potential use as a surrogate imaging marker to track PD progression in the longer-term. The poor correlation between putaminal MD and motor scores may again be affected by partial volume averaging effects between the anterior and posterior putamen in an automated putaminal mask (Wallis et al., 2008; Ulla et al., 2013; Chan et al., 2016), rather than incipient striatal floor effects described with dopamine transporter scans (Cilia et al., 2020).

Yang et al. (2018) suggested that PD-induced diffusion changes still require more follow-up validations to address the choice imaging markers for strategizing treatments in different phases of PD evolution. Most diffusion MRI studies in PD have only tracked changes up to 6 years (Chan et al., 2016; Yang et al., 2018; Sarasso et al., 2021). Recent longitudinal DTI studies on 3T scanners using similar standard DTI processing in early PD stages over 19.3 (18 PD; 14 HC) (Loane et al., 2016) and 12.6 months (120 PD; 50 HC) (Zhang Y. et al., 2016) employed manual and automated nuclear segmentation respectively. These studies reported negative striatal findings, congruent with our results in the first 6 years. However, our serial nuclear DTI data over 12 years show value-add in tracking disease progression on a longer-term basis, at the later stages of PD.

Our study has intrinsic limitations. First, interpretation of the FA changes in the putamen and thalamus is limited by the lack of iron-sensitive sequences in our MRI protocol. The first timepoint data collection (Chan et al., 2007) started before the advent of iron-sensitive quantitative techniques that are now widely accessible, and precluded inclusion for standardized study across all three timepoints. Future longitudinal DTI studies should include iron-sensitive sequences for accurate voxel-wise iron quantification to help dissect-out iron effects in the deep gray nuclei, besides long-term neuromodulatory effects of levodopa on iron chelation. Second, our DTI protocol is constrained by the assumption of free water diffusion, with attendant limitations. Again, this was the best technology available when data collection first began, (Chan et al., 2007) and remains the most accessible diffusion modeling, which we had applied consistently throughout the three timepoints to ensure comparability of data across the protracted study period. Future studies should leverage on higher-order diffusion MRI acquisitions and more sophisticated diffusion modeling to correct for the free-water fraction, such as the bi-tensor diffusion model (Ofori et al., 2015) or diffusion kurtosis imaging (Welton et al., 2023). These would better characterize the structural complexity and heterogeneities within the putamen and thalamus. Nonetheless, our serial deep gray nuclear DTI data over 12 years showed valid (Atkinson-Clement et al., 2017) and interesting findings that are substantiated by current literature (Xu et al., 2015; Du et al., 2018, 2022).

In conclusion, our longitudinal three timepoint MD changes over 12 years suggest progressive, differential striatal neurodegeneration related to motor disability in PD, consistent with concepts of sensorimotor basal ganglia-thalamocortical circuitry. FA changes were complex, and would require higher order diffusion modeling and multimodal longitudinal study to further evaluate the impact of iron and levodopa, and downstream neural reorganization. Temporal tracking of caudate diffusivity may be a helpful objective imaging marker following PD progression over the entire course of the disease across a decade.

## Data availability statement

The original contributions presented in this study are included in the article/Supplementary material, further inquiries can be directed to the corresponding author.

## Ethics statement

The studies involving human participants were reviewed and approved by the SingHealth Centralised Institutional Review Board (CIRB) CIRB2015/2587. The patients/participants provided their written informed consent to participate in this study.

## Author contributions

LC and ET contributed to the conception and design of the study. Y-CS, LO, H-HL, JA, SH, ET, and LC contributed to the acquisition and analysis of the data. Y-CS, LO, SH, TW, ET, and LC contributed to drafting of the manuscript. All authors contributed to the article and approved the submitted version.

## Footnotes

1
http://www.fmrib.ox.ac.uk/fsl

2
http://stnava.github.io/ANTs/
