# Supplementary material for: Serial deep gray nuclear DTI changes in Parkinson’s disease over twelve years
Source: Front Aging Neurosci. 2023 Jun 20;15:1169254. doi: 10.3389/fnagi.2023.1169254 (PMC10318173; doi:10.3389/fnagi.2023.1169254)
Supplement: Supplementary file 1 [file Data_Sheet_1.docx]

**Supplementary Materials**

**Different Coil Combination Modes**

Adjustments for potential nonuniform biases in DTI metrics arising from different coil combination modes with MRI console upgrading between baseline [sum-of-squares (SOS)], 2^nd^ timepoint [adaptive combination (AC)] and 3^rd^ timepoint (AC mode) scans was made by taking into account background noise floor and measured combined magnitude signals in a non-central-chi (ncΧ) probability distribution based tensor fit^1^ to reduce biases between DTI metrics derived from SOS and AC modes across the first two timepoints.

**Reference:**

1. Sakaie K, Lowe M. Retrospective correction of bias in diffusion tensor imaging arising from coil combination mode. Magn Reson Imaging. 2017 Apr;37:203-8.

**
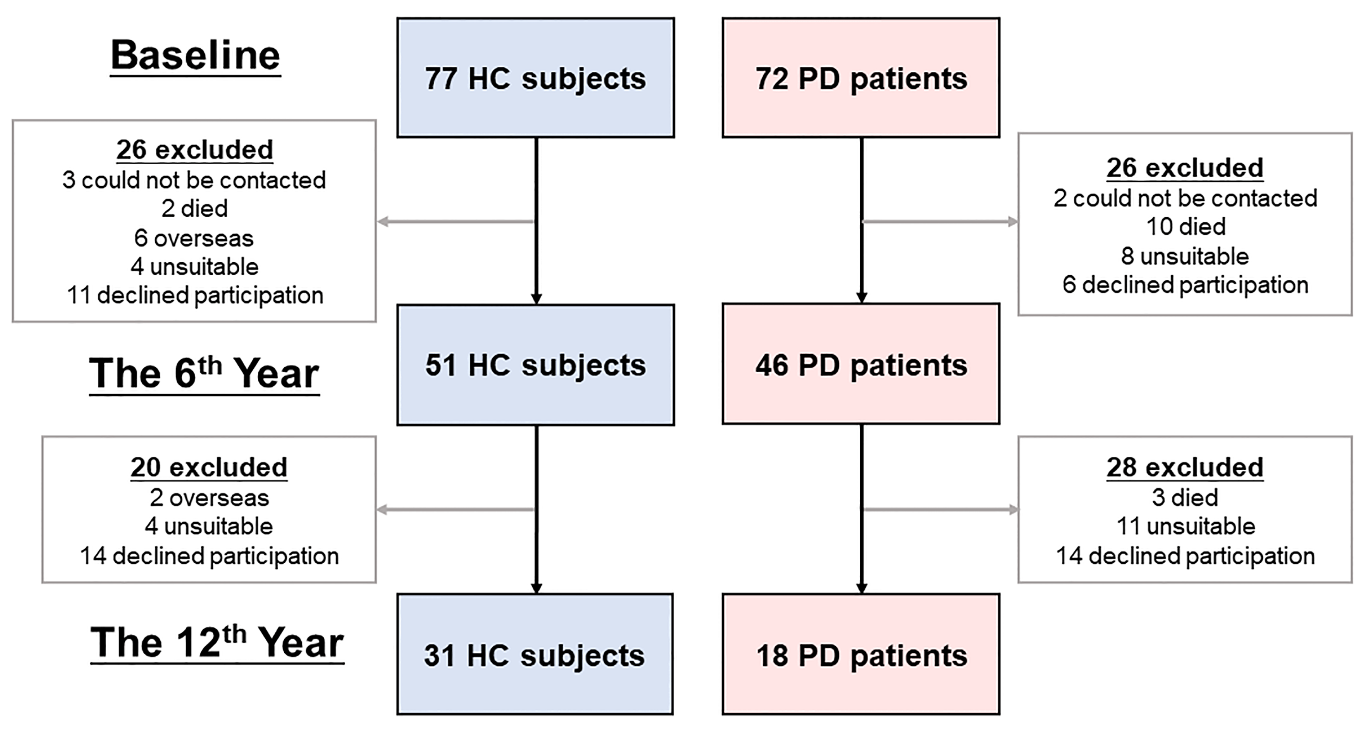
**

**Fig S1** Flow diagram showing the number of study participants in both healthy control (HC) and Parkinson’s disease (PD) groups from baseline (1^st^ timepoint) to the sixth- (2^nd^ timepoint) and twelfth-year (3^rd^ timepoint) MRI examinations in this longitudinal brain diffusion tensor imaging (DTI) MRI study. Two PD patients and three HC subjects were not contactable at 2^nd^ timepoint; but all subjects who returned for the 2^nd^ timepoint study were contacted at the 3^rd^ timepoint. Re-contacting subjects revealed that 13 PD patients (10 at 2^nd^ timepoint; 3 at 3^rd^ timepoint) and two HC (at 2^nd^ timepoint) had died (the cause of which was not available in all), and eight HC (6 at 2^nd^ timepoint; 2 at 3^rd^ timepoint) were living overseas. Nineteen PD patients (8 at 2^nd^ timepoint; 11 at 3^rd^ timepoint) and eight HC (4 each at 2^nd^ and 3^rd^ timepoints) were deemed unsuitable by the study team to return for brain MRI due to reasons such as MRI contraindications (including post deep brain stimulation surgery, pacemaker), advanced disease (Hoehn and Yahr [H&Y] stage 5), and new debilitating comorbidities (e.g., stroke, cancer, organ failure). Twenty PD patients (6 at 2^nd^ timepoint; 14 at 3^rd^ timepoint), and 25 HC (11 at 2^nd^ timepoint; 14 at 3^rd^ timepoint) declined participation due to multifactorial reasons including fractures, general frailty requiring assistance to travel, family commitments (looking after sick member or grandchildren) and frank refusal by subject or family.

**
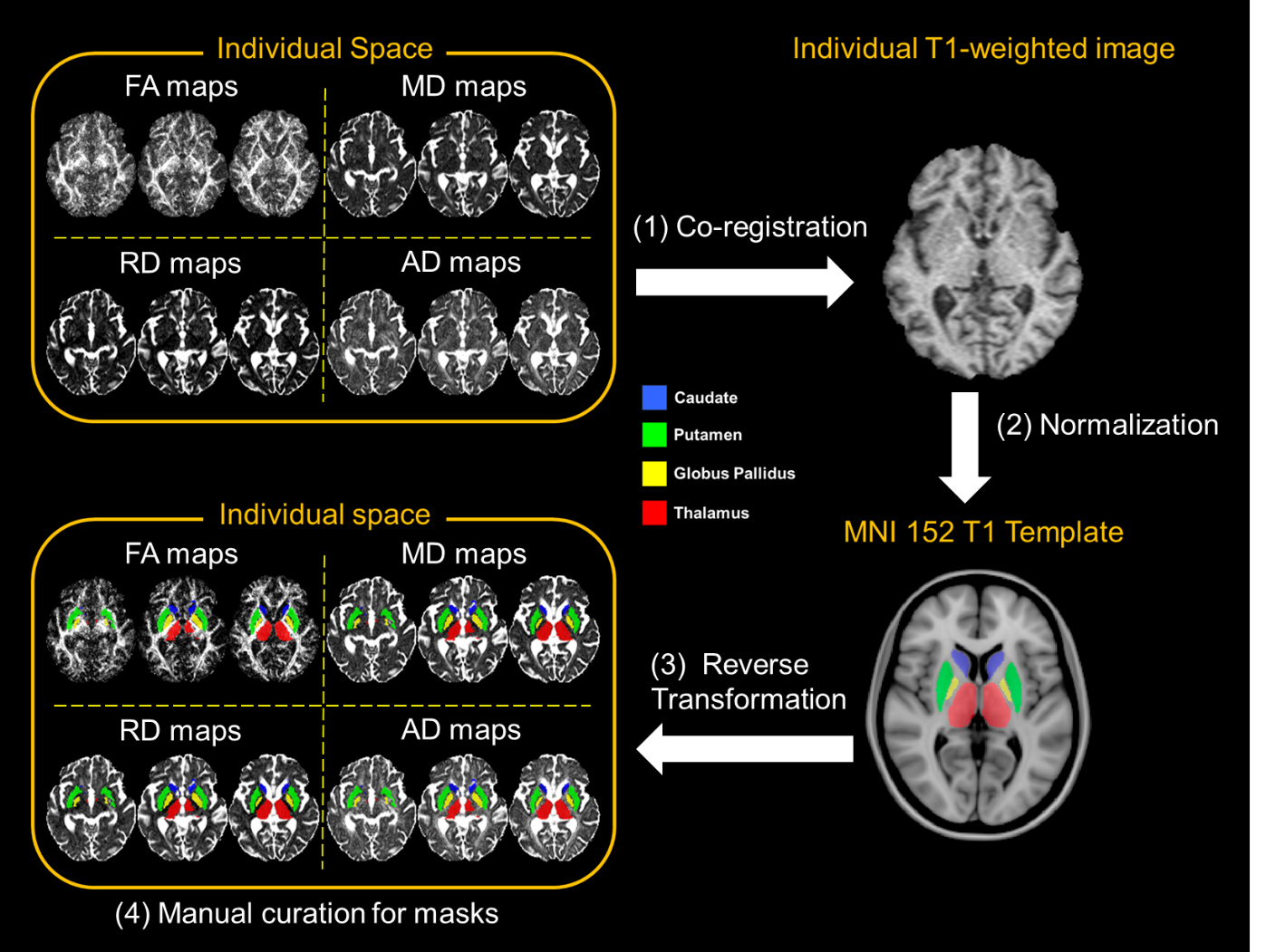
**

**Fig. S2** Schematic of semi-automated tissue segmentation pipeline. (1) All diffusion maps were co-registered to T1-weighted image in individual space for each subject using a rigid body transformation. (2) An individual T1-weigted image was normalized to a standard T1 template (MNI 152) using the Symmetric image Normalization method (SyN). Once the spatial normalization is done, we can obtain the transformation matrix between the MNI space and individual space. (3) The 8 masks of deep gray nuclei in both hemispheres, which were defined in the MNI space, were automatically reversely transformed back to the individual diffusion maps. Note that blue mask is caudate, green mask is putamen, yellow mask is globus pallidus, and red mask is thalamus. (4)

**Table S1** Comparison of clinical motor assessment in patients across the three timepoints (full cohort)

|  | **ANOVA** | **Tukey Honestly Significant Difference Post-Hoc Test** | | |
| --- | --- | --- | --- | --- |
|  | F(2,133) | 95% confidence interval | *p*-value | Hedges’ g |
| **H&Y staging** | 17.650 (*p* < 0.001) |  |  |  |
| 1st vs. 2nd timepoints |  | 0.046 – 0.674 | 0.020 | -0.5901 |
| 1st vs. 3rd timepoints |  | 0.641 – 1.519 | < 0.001 | -2.2895 |
| 2nd vs. 3rd timepoints |  | 0.257 – 1.1830 | 0.001 | -0.8317 |
| **UPDRS-III** | 28.760 (*p* < 0.001) |  |  |  |
| 1st vs. 2nd timepoints |  | 1.725 – 9.115 | 0.002 | -0.7534 |
| 1st vs. 3rd timepoints |  | 11.041 – 21.359 | < 0.001 | -1.8878 |
| 2nd vs. 3rd timepoints |  | 5.338 – 16.222 | < 0.001 | -1.1279 |

Abbreviations: H&Y = Hoehn and Yahr staging, UPDRS = United Parkinson Disease Rating Scale.
